# Environmental Chemicals and Maternal Depression During and After Pregnancy: a Scoping Review

**DOI:** 10.1007/s40572-026-00529-7

**Published:** 2026-02-19

**Authors:** Pengfei Guo, Yunyue Shi, Cindy Nguyen, Haoran Zhuo, Tormod Rogne, Zeyan Liew

**Affiliations:** 1 Department of Epidemiology and Biostatistics, Arnold School of Public Health, University of South Carolina, 915 Greene Street, Columbia, SC 29208 USA; 2 Department of Environmental Health Sciences, Yale School of Public Health, New Haven, CT USA; 3 Yale Center for Perinatal, Pediatric, and Environmental Epidemiology, Yale School of Public Health, New Haven, CT USA; 4 Department of Cellular, Molecular, and Developmental Biology, Yale College, New Haven, CT USA; 5 Department of Community Medicine and Global Health, University of Oslo, Oslo, Norway; 6 Department of Chronic Disease Epidemiology, Yale School of Public Health, New Haven, CT USA

**Keywords:** Reproduction, Mental disorders, Environmental pollution, Organic chemicals, Inorganic chemicals

## Abstract

**Purpose of Review:**

There is increasing evidence that several environmental exposures may pose a risk for depression, including maternal depression. We conducted a scoping review of epidemiological evidence regarding maternal exposure to environmental chemicals and perinatal depression.

**Recent Findings:**

We searched PubMed, Embase, Web of Science, Dimensions, and Scopus, and summarized the findings from 27 articles that examined environmental chemical exposures and maternal depression. Studies of ambient air pollutants (*N* = 11) showed exposure to NO_2_ and PM_10_ to be most consistently associated with antenatal or postnatal depression. Studies of endocrine-disrupting chemicals, including phthalates (*n* = 6), per- and polyfluoroalkyl substances (PFAS, *n* = 6), polybrominated diphenyl ethers (PBDE, *n* = 3), organophosphate esters flame retardants (OPE, *n* = 2), and pesticides (*n* = 1), reported positive links with maternal depression, particularly from exposures to phthalates and PBDE. Studies of the individual and mixture of metals (*n* = 3) have reported mixed results.

**Summary:**

Maternal exposures to certain airborne pollutants, and chemicals from contaminated household products and food sources, are associated with maternal depression. If these findings are confirmed, reducing environmental risks may represent a promising strategy for the primary prevention of maternal depression.

**Supplementary Information:**

The online version contains supplementary material available at 10.1007/s40572-026-00529-7.

## Introduction

 Depression affects approximately 10–20% of women during pregnancy and the first year following delivery [1, 2]. The prevalence has been reported to be higher in low- and middle-income countries [3]. Maternal perinatal depression has been an overlooked complication of childbearing [3], often characterized by a range of severe and persistent symptoms of depression, anxiety, and emotional stress. Untreated maternal depression is associated with a spectrum of adverse outcomes during pregnancy and after delivery [4]. It increases the likelihood of obstetric complications and unfavorable birth outcomes, disrupts breastfeeding, and exacerbates sleep disturbance, sexual function, substance use, and medication reliance [4]. Over the life course, impaired mental health during pregnancy and postpartum heightens the susceptibility to chronic diseases, disability, and premature mortality in mothers [4, 5]. Mothers experiencing depressive symptoms often have low maternal functioning, and their children are more likely to experience altered neurodevelopment and poor social-emotional well-being [6–8].

The etiology of maternal depression is multifactorial and not fully understood [9, 10]. Genome‑wide association studies have not identified any major‑effect loci, underscoring the importance of considering the role of environmental factors in the etiology of maternal depression [11]. Biologically, the substantial fluctuations in reproductive and stress hormone levels during pregnancy, including estrogen, progesterone, and cortisol, followed by their abrupt withdrawal postpartum, are thought to predict maternal depressive symptoms [12]. These hormonal shifts may dysregulate brain dopaminergic pathways and the hypothalamic-pituitary-adrenal (HPA) axis, a central neurobiological pathway involved in the pathophysiology of depression [13]. Environmentally induced chronic inflammation and oxidative stress-mediated neuropathology [10] can also influence depression. Major psychosocial stressors, such as severe life events, poor relationships, and limited family support, are well-established predictors of poor mental health during the perinatal period [10].

Recently, many environmental pollutants such as airborne pollutants, endocrine-disrupting chemicals (EDC), and metals have been suggested as neurotoxicants that may affect depression development through oxidative, inflammatory, endocrine, and epigenetic modifications [14–16]. Exposures to ambient nitrogen dioxide and ozone can trigger reactive oxygen species (ROS) production, systemic cytokine responses, and downstream HPA-axis stimulation leading to acute or chronic depression-like behaviors [17, 18]. Particulate matter can cross the air–blood barrier, inducing hippocampal pro-inflammatory cytokine expression, synaptic pruning, and depression-like phenotypes [19, 20]. Perturbation of hormonal pathways represents a plausible mechanism for EDC exposures [21, 22]. For example, phthalates may exaggerate hypothalamic neuron activity, disrupt brain lipidomes, and produce persistent depressive-like traits [23]; bisphenol A can disturb neurotransmitter and neuroactive-steroid homeostasis and increase corticosterone-dependent despair in rodents [24]. Phthalates and BPA exposures are also linked to altered epigenetic outcomes in the human placenta, including microRNA expression, DNA methylation, and genomic imprinting [25]. Transition-metal toxicants can accumulate in the limbic system that controls emotions, memory, and stress responses, e.g., cadmium initiates neuroinflammation [26] or lead increases lipid peroxidation and reduces antioxidants [27] that in turn cause anxiety- and depression-like behaviors. Although evidence of environmental exposure risks for depression is accumulating, studies that focus on pregnant and postpartum populations remain sparse [28].

We performed a scoping review of epidemiological evidence regarding the effects of environmental chemical exposures on maternal depression, encompassing depressive, anxiety, and psychological stress symptoms. By mapping current knowledge, this review aimed to identify research gaps and propose priorities for future investigations in this evolving field.

## Methods

We followed the checklist of the Preferred Reporting Items for Systematic Reviews and Meta-Analyses extension for Scoping Reviews [29]. We searched PubMed, Embase, Web of Science, Dimensions, and Scopus for literature published up to August 2024, with no restriction on start year. As the goal was to summarize peer-reviewed epidemiological original research articles that focused on examining environmental chemical exposures, the following studies were excluded: (1) experimental studies (non-human population-based); (2) studies evaluating other types of environmental factors, such as light, noise, radiation, vibration, behavioral factors (active smoking, alcohol consumption, diet, nutrition, medication, or treatment), infection of biological agents including viruses; (3) other pregnancy outcomes such as obstetric complications, birth outcomes, or offspring health, (4) mental health outcomes other than depression, anxiety, or stress, or outcomes unrelated to pregnancy (5) other types of documents, such as research protocol, review papers, commentaries, conference abstracts, dissertations, and case studies. We did not limit the search to certain geography, demography, or assessment tools for either exposure or outcome. We did not place language restrictions on the search. We also did not exclude studies based on the length of the postpartum period investigated, instead, we documented the definitions for each study that explicitly aimed to investigate postpartum depression. In the main text, we focused on discussing studies that assessed depression, while details for anxiety and emotional stress are presented in the supplemental materials (Tables S1−2).

The searches were tailored to each database and are presented in Appendix A. The last access date for database searches was August 16, 2024. We used Boolean operators (OR, AND) and truncation symbols to optimize the search scope as needed. First, two researchers (authors YS and CN) independently screened the titles and abstracts for articles on the Covidence review platform (covidence.org). The two researchers independently extracted the key characteristics of each article, which were then reviewed in full-text [30], including study location and duration, design and population, exposure and outcome measurement, statistical approach, and main findings. Discrepancies in screening results and in the data extraction were discussed and determined by the lead author (PG). No protocol registration (e.g., OSF or PROSPERO) was conducted.

## Results and Discussion

### General Study Characteristics

**Fig. 1:**
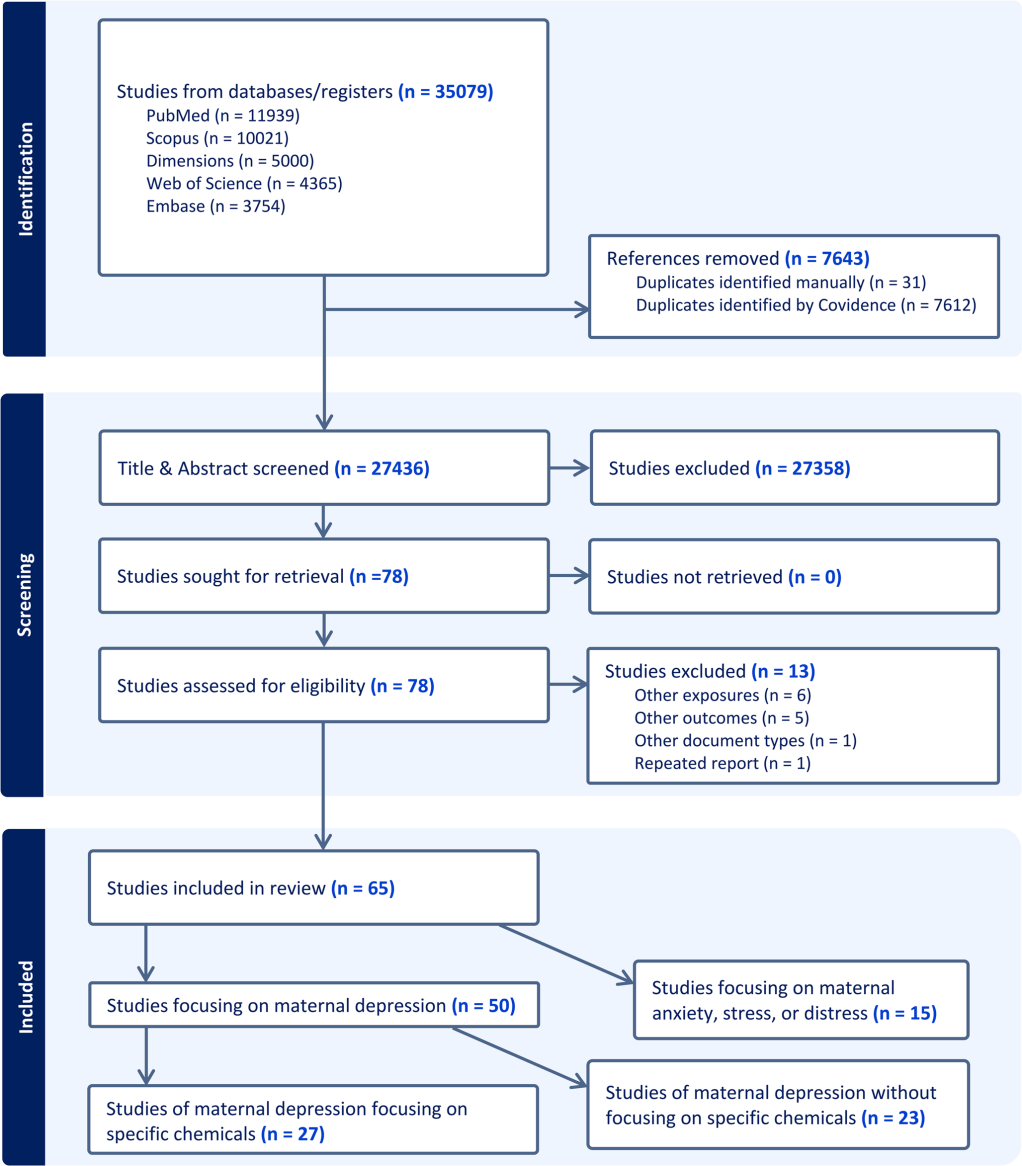
PRISMA Flow diagram depicting the process of article selection. ^a^ WOS: Web of Science Core Collection as licensed at Yale Institution

Figure 1 displays the PRISMA flowchart of study inclusion and exclusion in this review. Seventy-eight full-text articles were assessed for eligibility, with 13 excluded because they focused on ineligible exposures (*n* = 6), outcomes (*n* = 5), document type (*n* = 1), or repeated secondary report (*n* = 1). In total, 65 articles met our eligibility criteria. Over two-thirds of the studies were published relatively recently between 2021 and 2024 (Figure S1). Among these studies, 50 studies examined maternal depression, with others focusing on maternal anxiety, emotional stress, or distress. Maternal depression was primarily assessed by maternal reports using validated instruments, with few (*n* = 4) studies based on medical records or registries that tracked maternal clinical diagnosis records. The majority of the reported study design was cohort (*n* = 39), followed by cross-sectional (*n* = 24) and case-control (*n* = 2). The sample sizes ranged from 50 to 340,679, with the largest studies on ambient air pollution using geospatial models and maternal residential addresses. The studies relying on biological samples, measuring metals or endocrine-disrupting chemicals (EDC), were generally smaller with the largest sample size analyzed up to 2,174. The most used screening instruments for depressive symptoms included the Edinburgh Postnatal Depression Scale (EPDS) [31], the Center for Epidemiologic Studies Depression Scale (CES-D) [32], and the Beck Depression Inventory-II (BDI-II) [32]. Most studies screened maternal depression up to one year after delivery (if not otherwise specified in this review), with repeated screenings conducted in some of the studies at different months postpartum. The key study characteristics are presented in Tables S1−2.

Overall, 19 studies examined maternal residential ambient air pollution, 17 on EDC, and 9 on metals. Studies of indoor air quality (*n* = 3), environmental tobacco smoke (ETS) (*n* = 16), and unconventional oil and gas development (*n* = 1) are not specifically discussed below because they did not measure or detail the specific type of chemicals assessed. However, the summary of the study characteristics and findings can be found in Table S2. In the following sections, we summarize the results for 27 studies on depression that examined maternal residential ambient air pollution (*n* = 11), EDC (*n* = 13), and metals (*n* = 3).

### Ambient Air Pollution

The eleven studies of air pollution and maternal depression are listed in Table 1. These studies had focused on examining the criteria air pollutants, assessed with geospatially estimated exposures linked to maternal residential addresses. Criteria air pollutants refer to a group of major outdoor air contaminants, including particulate matter (PM), carbon monoxide (CO), ozone (O_3_), nitrogen dioxide (NO_2_), and sulfur dioxide (SO_2_). They are regulated due to their health risks, and they commonly originate from sources such as traffic emissions, industrial activities, fossil fuel combustion, and climate events like wildfires and dust storms [44, 45]. Corroborating with findings from a recent meta-analysis [35, 38, 41, 46], our review found that PM_10_ (PM with a diameter of 10 μm or smaller) and NO_2_ were consistently reported to be associated with increased maternal depression diagnoses or symptoms [34, 35, 38–41]. PM_2.5_ (PM with diameters of 2.5 μm or smaller) and its chemical components, such as black carbon and organic matter, were also reported to be associated with depression in pregnancy, but two studies reported null results for depression at one-month [42] or six-month postpartum [41]. Two studies linked SO_2_ exposure to higher depressive symptoms or diagnosed depressive disorder during and post-pregnancy [35, 39]. The associations for CO and O_3_ were inconclusive with some decreased odds of depression in pregnancy reported in two studies [39, 40]. Inconclusive results may largely be due to differences in sample sizes, study populations, outcome measurement timing, and study regions with different compositions or levels of PM_2.5_. A few studies that examined susceptible windows of air pollution exposures reported exposures in the first [36, 41] and second trimesters [38, 40, 43] to be important for maternal depression. Other than depression, outdoor NO_2_ or PM_2.5_ exposure was also reported in some studies to be associated with higher odds of anxiety [33, 47], stress [37, 48–50], and maternal annoyance [51].

**Table 1:** Summary of articles examining the associations between ambient air pollution and maternal depression

Study	Study design	Study period	Study location	Case/Sample size	Exposure assessment	Outcome assessment	Key findings
Qiu [33]	Cross-sectional	Mar 2019–Oct 2021	Wuhan, China	37/75	PM2.5: extract daily levels from the TAP database at residential addresses and use a two-stage machine learning algorithm; PM2.5 chemical components: use the WRF-CMAQ model, collected in TRI-3 (gestation 28–40 weeks, average gestation 35.86 weeks)	AD (EPDS score > = 10), collected in TRI-3 (gestation 28–40 weeks, average gestation 35.86 weeks)	+: Short-term exposure to PM2.5 and its chemical components (OM and BC) were positively associated with AD (lag 0–30: PM2.5 OR = 1.15, 95%CI: 1.04, 1.27; OM OR = 1.46, 95%CI: 1.04, 2.05; BC OR = 7.93, 95%CI: 1.19, 52.64).
Sun [34]	Cohort	2008–2016	Southern California, USA	25,674/340,679	Based on maternal residential addresses, monthly averages of PM2.5, PM10, NO2, and O3 collected from spatial interpolation of monitoring station measurements; Constituents of PM2.5 obtained from fine-resolution geoscience-derived models based on satellite, ground-based monitor, and chemical transport modeling data; averaged for monthly, trimester-specific, and postpartum exposures	PPD (EPDS score > = 10), using a combination of diagnostic codes and prescription medications, clinical interviews, ICD codes, and pharmacy records in KPSC EHRs during 6 months postpartum	+: Long-term antepartum and postpartum exposures to O3 (OR = 1.09, 95%CI: 1.06, 1.12), PM10 (OR = 1.02, 95%CI: 1.00,1.04), and PM2.5 (OR = 1.02, 95%CI: 1.00, 1.03) and its chemical constituents (OM and BC) were associated with an increased risk of PPD.
Duan [35]	Cohort	Oct 2019–Feb 2021	Shanghai, Hangzhou, Shaoxing, China	EPDS > = 10: 2182/10,209; EPDS > = 13: 1087/10,209	PM2.5, PM10, SO2, CO, NO2, and O3 data from the National Monitoring Center averaged for each city, averaged over the whole pregnancy and each trimester	PPD (EPDS score > = 10 and > = 13) at 6 weeks postpartum	+: A 10 µg/m3 increase in pregnancy-averaged PM10 (OR = 1.47, 95% CI:1.36, 1.59), NO2 (OR = 1.63, 95%CI: 1.44, 1.85), and 0.1 mg/m3 increase in CO (OR = 2.31, 95% CI: 1.99, 2.69) increased the risk of PPD symptoms, with similar results found in each trimester. TRI-2 SO2 exposure was a major risk factor for developing PPD symptoms (OR = 1.10, 95% CI: 1.03, 1.18).
Zhao [36]	Cohort	Jun 2016– May 2018	Shanghai, China	420/3731	PM2.5: Gap-filling random forest model based on satellite data, assigned according to the maternal residential of each birth record, mean exposure during pregnancy was calculated for 3 gestational periods (0–13 weeks, 0–26 weeks, and 0–36 weeks)	AD (CES-D score > = 16) during late pregnancy (32–36 weeks)	+: Increased PM2.5 during pregnancy was associated with prenatal depression, especially in early pregnancy. Each 10 µg/m3 increase in PM2.5 was associated with a β of 0.510 (95%CI: 0.22, 0.80) for CES-D scores. Each 10 µg/m3 increase in PM2.5 was associated with an increased risk of depression by approximately 25%.
Ahlers [37]	Cross-sectional	2015–2019	San Francisco Bay Area, California, USA	NA/50	Estimated from the nearest air quality monitoring station based on maternal residential area; calculated four air pollution measures for the analyses, including the average PM2.5 exposure over pregnancy and estimates for each clinically defined trimester (TRI-1: 1–13 weeks, TRI-2: 14–27 weeks, TRI-3: 28 weeks-delivery)	Depression (PHQ-9 score > 10) assessed during the 3rd trimester	Increased prenatal exposure to PM2.5 across pregnancy was associated with more severe depressive symptoms during the TRI-3 (β = 0.14, *p* = 0.02).
Bastain [38]	Cohort	2015–2020	Los Angeles, CA, USA	29/180	NO2, O3, PM2.5, PM10: used inverse-distance squared spatial interpolation from ambient monitoring data, averaged for each trimester and across pregnancy	PPD (CES-D score > = 16) at 12 months postpartum	+: Over two-fold increased odds of 12 months PPD were associated with TRI-2 NO2 (OR = 2.63, 95%CI: 1.41, 4.89) and pregnancy-average NO2 (OR = 2.04, 95% CI: 1.13, 3.69). Higher TRI-2 PM2.5 was associated with increased 12 months PPD (OR = 1.56, 95%CI: 1.01, 2.42). The effect for TRI-2 PM10 was similar and was borderline significant (OR = 1.58, 95% CI: 0.97, 2.56).
Kanner [39]	Cohort	2002–2008	USA	Any: 9793/221,794 Only: 4862/221,794	PM10, PM2.5, CO, NO2, NOx, SO2, O3: derived from a modified Community Multiscale Air Quality mode during 3-months preconception, TRI-1, and whole pregnancy	Any depression: diagnosis of major depressive disorder (ICD9 296.2, 296.3) or depressive disorder not elsewhere classified (ICD9 311) in discharge summaries, or women with a history of depression recorded in electronic medical records; Only depression: no other mental health conditions	+: Whole pregnancy exposure to PM10, PM2.5, NO2, and NOx was associated with 11%–21% increased odds of any depression. -: CO and O3 were associated with 16%–20% decreased odds of any depression. +: Whole pregnancy exposure to PM10, NO2, and SO2 was associated with 18%−151% increased odds of only depression. -: PM2.5, CO, NOx, and O3 were associated with 31%−49% decreased odds of only depression.
Lamichhane [40]	Cohort	2008–2015	Seoul Metropolitan area, South Korea	NA/1481	Monthly PM2.5, PM10, NO2, and O3 exposure at residential addresses using LUR models, assessed for each trimester and the entire pregnancy	AD (CES-D-10 score > = 12) assessed in TRI-3	+: An IQR increase in TRI-2 PM2.5 (RR = 1.15, 95%CI: 1.04, 1.27), PM10 (RR = 1.13, 95%CI: 1.04, 1.23), and NO2 (RR = 1.15, 95%CI: 1.03, 1.29) and TRI-3 O3 (RR = 1.09, 95%CI: 1.01, 1.18) were associated with increased risks of TRI-3 depressive symptoms. **-**: An IQR increase in TRI-1 O3 was associated with a decreased risk of depressive symptoms (RR = 0.89, 95%CI: 0.82, 0.96).
Shih [41]	Cohort	2005	Taiwan, China	3648/21,188	PM2.5 and NO2 estimated using a hybrid kriging/LUR model, and CO exposure estimated using a LUR-based extreme gradient boosting algorithm model based on data from the air monitoring stations, assessed in the TRI-1 (conception–12th week), TRI-2 (13–26 week), TRI-3 (27 week+), and 3 months postpartum	Self-reported by mothers via a questionnaire regarding whether they experienced PPD within 6 months postpartum	+: Exposure to IQR, namely, 10.67 ppb higher in NO2 in TRI-1, was associated with an 8% increase in the likelihood of PPD (OR = 1.08, 95%CI: 1.00, 1.16), but **not** to PM2.5 or CO.
Niedzwiecki [42]	Cohort	Jul 2007–Feb 2011	Mexico City	During pregnancy: 136/509, 1-month PPD: 93/509, 6 months PPD: 90/509	Hybrid satellite-based spatio-temporally resolved model and averaged over pregnancy and the first year postpartum, estimated daily residential PM2.5 levels for each participant	Probable PPD (EPDS score > = 13) at 1 month and 6 months postpartum	**Null**: PM2.5 was not associated with 1-month PPD (RR = 0.85, 95%CI: 0.61, 1.19). +: A 5-µg/m3 increase in average PM2.5 exposure during pregnancy was associated with an increased risk of 6 months PPD (RR = 1.59, 95%CI: 1.11, 2.28) and of late-onset PPD (PPD at 6 months with no PPD at 1 month) (RR = 2.58, 95%CI: 1.40, 4.73).
Sheffield [43]	Cohort	2002–2007	Boston, MA, USA	NA/557	Using a satellite-based spatio-temporally resolved model, women’s gestational exposure estimates were calculated by averaging PM2.5 daily predictions over each week of pregnancy, over gestation	EPDS total scores at 6 and 12 months postpartum via face-to-face interviews; higher scores from either time used for analysis	+: Increased TRI-2 PM2.5 exposure was associated with increased depressive and anhedonia symptoms.

Abbreviations: *PPD* postpartum depression, *AD* antenatal depression, *TRI-1* 1 st trimester, *TRI-2* 2nd trimester, *TRI-3* 3rd trimester, *OR* odds ratio, *RR* risk ratio, *CI* confidence interval, *IQR* interquartile range, *EPDS* Edinburgh Postnatal Depression Scale, *CES-D* Center for Epidemiologic Studies Depression Scale, *PHQ-9* the 9 item Patient Health Questionnaire, *LUR* land use regression, *OM* organic matter, *BC* black carbon, *PM2.5* particulate matter less than or equal to 2.5 μm, *PM10* particulate matter less than or equal to 10 μm, *NO2* nitrogen dioxide, *NOx,* nitrogen oxide, *O3* ozone, *SO2* sulfur dioxide, *CO* carbon monoxide, *TAP* Tracking Air Pollution in China, *WRF-CMAQ* Weather Research and Forecasting-Community Multiscale Air Quality, *KPSC EHRs* Kaiser Permanente Southern California electronic health records, *ICD* International Classification of Diseases, *ICD9* International Classification of Diseases − 9.

In summary, particulate matter and NO₂ were most consistently associated with increased maternal depression during pregnancy and postpartum, while the results for SO₂, CO, and O₃ were not yet conclusive. Air pollution was suggested to influence maternal mental health starting from early pregnancy, underscoring the importance of early screening and intervention during pregnancy, particularly in areas affected by elevated pollution levels.

### Endocrine-Disrupting Chemicals (EDC)

Table 2 presents the study characteristics of the 13 studies on maternal depression in relation to a variety of EDC exposures. Environmental phthalates, phenols (bisphenols and triclosan), and parabens are known or suspected prevalent and nonpersistent endocrine disruptors [65]. They are widespread due to their softening effect or serving as preservatives and additives in a variety of personal care and consumer products [66]. Per- and polyfluoroalkyl substances (PFAS) are a large group of synthetic fluorinated compounds widely used in industrial and commercial applications, including kitchenware, food packaging, clothing, and carpeting [67]. Polybrominated Diphenyl Ethers (PBDE) are flame retardants used for furniture and carpet padding and in the hard plastic casings in electronics and appliances [68]. Organophosphate esters (OPE) flame retardant chemicals have increased in use after PBDE were phased out due to toxicity concerns in the early 2000 s [69]. Organochlorine pesticides (OCP), once extensively applied in agriculture and vector control worldwide, are effective neurotoxicants to insects but are also characterized by long-lasting residues in the environment [70].

**Table 2:** Summary of articles examining the associations between endocrine-disrupting chemicals and maternal depression

Citation	Study design	Study year	Location	Sample size	Exposure measurement	Outcome measurement	Key findings
Foster [52]	Cohort	2008–2012	Edmonton, Toronto, Vancouver, Winnipeg/Morten-Winkler, Canada	18 weeks’ gestation: 118/718, 1 year postpartum: 100/718	GC-MS/MS for 29 OPEs using house dust samples at 3–4 months postpartum; created total OPE Z-score (∑OPE of 14 OPEs for analysis)	PND (continuous CES-D score) at 18- and 36-weeks’ gestation and 6 months and 1 year postpartum; CES-D score > = 16 used in supplementary model	**Null**: OPEs were not associated with CES-D scores.
Hernandez-Castro [53]	Cohort	2015–2023	Los Angeles, California, USA	137/422	HPLC-QTRAP 5500 + MS/MS for 10 OPEs (BBOEP, BCEP, BCIPP, BDCIPP, BEHP, BMPP, DNBP, DIBP, DPHP, DPRP) using urine samples collected in TRI-3 (31.5 ± 2.0 weeks gestation)	AD (CES-D score > = 16) during pregnancy	+: Participants with the highest tertiles of DPHP and BDCIPP exposure had a 67% (95%CI: 22%, 128%) and 47% (95%CI: 4%, 108%) increased risk of AD, respectively. **Null**: No associations between other OPE metabolites and AD were observed. +: A linear association between higher exposure to OPE metabolite mixture (BBOEP, BCEP, BDCIPP, DNBP + DIBP, DPHP) and odds of AD was detected, primarily driven by DPHP (OR = 1.28, 95%CI: 1.06, 1.55).
Yalçın [54]	Cohort	NA	Suburban area of Ankara, Turkey	NA/75	SPE-GC-ECD for 12 OCPs (α-HCH, β-HCH, γ-HCH, aldrin, dieldrin, heptachlor, heptachlor epoxide, α-endosulfan, β-endosulfan, trans-chlordane, cis-chlordane, DDT) using human milk samples collected at 8 months postpartum	PPD (continuous EPDS scores used in correlation analyses) at 8 months postpartum	**Null**: No relation was detected between EPDS and OCPs.
Hu [55]	Cohort	March 2016– Jan. 2018	Wuhan, China	PND during pregnancy: 50/150; PND at 1 month postpartum: 51/143; PND at 6 months postpartum: 37/125	HPLC-MS/MS for 8 PFAS (PFOA, PFDA, PFNA, PFUdA, PFOS, PFHxS, PFHpS, 6:2 Cl-PFESA) using maternal plasma collected at each visit (median = 13-, 24-, and 30-weeks’ gestation)	PND (Chinese version EPDS score > = 10) at early pregnancy and at 1 and 6 months postpartum	+: PFAS were individually and jointly associated with a higher risk of PND at 6 months but **not** at 1 month postpartum. A quartile increase in the PFAS mixture during the 1 st, 2nd, 3rd, and average pregnancy was associated with a RR of 1.73 (95%CI: 1.42, 2.12), 1.54 (95%CI: 1.27, 1.84), 1.75 (95%CI: 1.49, 2.08), and 1.63 (95%CI: 1.35, 1.97) for PND at 6 months, respectively. TRI-1 PFHxS (RR = 1.29, 95%CI: 1.02, 1.62), TRI-2 PFOS (RR = 1.16, 95%CI: 1.00, 1.35), and average PFHxS during pregnancy (RR = 1.34, 95%CI: 1.00, 1.79) was associated with PND at 1 month. TRI-1 PFNA (RR = 1.48, 95%CI: 1.09, 2.02), PFDA (RR = 1.34, 95%CI: 1.06, 1.69), PFHxS (RR = 1.38, 95%CI: 1.03, 1.84), TRI-3 PFOA (RR = 1.38, 95%CI: 1.00, 1.91) and PFHxS (RR = 1.40, 95%CI: 1.01, 1.92), and average PFDA (RR = 1.42, 95%CI: 1.03, 1.96) and PFHxS (RR = 1.53, 95%CI: 1.02, 2.29) during pregnancy was associated with PND at 6 months.
Aung [56]	Cross-sectional	NA	San Francisco, California, USA	42/521	SPE-LC-MS/MS for 12 PFAS (PFBS, PFHxS, PFOS, PFHpA, PFOA, PFNA, PFDeA, PFUdA, PFDoA, PFOSA, Me-PFOSA-AcOH, Et-PFOSA-AcOH) using serum samples collected in TRI-2 (12–28 weeks’ gestation)	AD (CES-D score > = 16) in TRI-2; continuous CES-D score used in regression models	+: PFAS are associated with higher AD among immigrant women during pregnancy (PFOS β = 2.7, 95%CI: 0.7, 4.7 and Me-PFOSA-AcOH β = 2.9, 95%CI: 1.2, 4.7). PFAS mixture was associated with increased AD among immigrant women (β = 1.12, 95%CI: 0.002, 2.3) compared to US-born women (β = 0.09, 95%CI: −1.0, 0.8).
Wang [57]	Cohort	2013–2016	Shanghai, China	327/2741	HPLC-MS/MS for 10 PFAS (PFOA, PFOS, PFNA, PFDA, PFUA, PFHxS, PFHpA, PFBS, PFDoA, PFOSA) using blood samples collected during TRI-1 (12.5 ± 1.8 gestational weeks); categorized into 3 tertiles	Probable PPD (EPDS score > = 10) at 6 weeks postpartum	**Null**: Exposure to most PFAS in early pregnancy was not associated with PPD. PFAS mixture was not associated with PPD (OR = 1.08, 95%CI: 0.91, 1.29). The middle tertile of PFDoA and the upper tertile of PFDoA were associated with 1.66-fold (95%CI: 1.21, 2.28) and 1.54-fold (95%CI: 1.10, 2.14) increased risk of PPD. The higher tertile of PFBS and PFHpA were associated with an increased risk of PPD, while the middle tertile of PFDA was associated with a decreased risk of PPD (OR = 0.72, 95%CI: 0.54, 0.96).
Jacobson [58]	Cohort	2006–2020	USA and Puerto Rico	Sensitive PPD: 349/2174 Specific PPD: 170/2174	HPLC-MS/MS for 2 bisphenols, 12 phthalate metabolites, 3 parabens, TCS, triclocarban using urine samples collected during pregnancy	Sensitive PPD (EPDS score > = 10 and CES-D score > = 16); Specific PPD (EPDS score > = 13 and CES-D score > = 20), assessed between 2 weeks and 12 months postpartum; EPDS and CES-D scale harmonized to the PROMIS depression scale.	**Null**: No associations between any chemical exposures and PROMIS depression T scores. +: LMW and HMW phthalates and DEHP had estimates in the positive direction, whereas all others were **negative**. +: HMW phthalates were associated with increased odds of sensitive PPD (OR = 1.11, 95%CI: 1.00, 1.23) and specific PPD (OR = 1.10, 95%CI: 0.96, 1.27).
Hu [59]	Cohort	Mar 2016 – Oct 2018	Wuhan, China	Early pregnancy: 95/278 1-month postpartum: 78/231 6 months postpartum: 52/182	LLE-ID-HPLC-MS/MS for 14 bisphenols, parabens, and phthalates (BPA, BPF, BPS, MeP, EtP, PrP, MEP, MiBP, MBP, MBzP, MEHP, MEHHP, MEOHP, MECPP) using urine samples collected each trimester (12–16 weeks, 16–28 weeks, and 28–42 weeks of gestation) (median = 13, 24, and 30 weeks of gestation)	PND (EPDS score > = 10) at early pregnancy and 1 and 6 months postpartum	+: One quartile increase in the TRI-2 chemical mixture (bisphenols, parabens, and phthalate metabolites) was associated with a 1.03-point (95%CI: 0.07, 1.99) higher 1-month PND, in which BPA and BPF contributed the most to the positive association. TRI-2 BPA and BPF showed the strongest associations with increased PND, and some of the associations differed across trimesters.
Jacobson [60]	Cohort	2016–2018	New York, USA	PPD: 12/139 AD: 20/139	HPLC-MS/MS for 8 bisphenols (BPA, BPAF, BPAP, BPB, BPF, BPP, BPS, BPPZ) and 22 phthalate metabolites using urine samples in early (< 18 weeks) and mid-pregnancy (> = 18–<25 weeks)	PPD (EPDS score > = 10) at 4 months postpartum	**Null**: There were no statistically significant associations with either bisphenols or phthalates. +: ∑DnOP was associated with increased odds of PPD (OR = 1.48, 95%CI: 1.04, 2.11).
Kim [61]	Cohort	Jul to Sep 2018	South Korea	95/221	LC-MS/MS using breast milk to analyze 5 phenols (BPA, MP, EP, PP, TCS) and 10 phthalate metabolites within 4 weeks of childbirth. 14 PFAS were measured by a Vanquish UPLC system coupled to a TSQ Quantis MS.	PPD (EPDS score > = 9) within the last 7 days, collected along with breast milk	**Null**: EDCs were not associated with maternal PPD.
Peltier [62]	Case-control	2013–2017	California, USA	22/367	GC-MS/MS for PBDE-47 using maternal plasma at 8–14 weeks’ gestation	PPDS (ICD-10 diagnosis) up to 1 year postpartum	+: Each two-fold increase in PBDE-47 concentrations increased the risk of PPD by 22% (OR = 1.22, 95%CI: 1.02, 1.49).
Mutic [63]	Cross-sectional	2014–2017	Georgia, USA	52/193	GC-MS/MS for PBDE − 47, −85, − 99, −100, − 153, and − 154 and mixture (− 47, − 99, −100) using maternal serum at 8–14 weeks’ gestation	AD (EDS score > = 10) at 8–14 weeks gestation	+: PBDE 47 (OR = 4.43, 95%CI: 1.47, 13.40) and PBDE 99 (OR = 1.58, 95%CI: 1.08, 3.00) were associated with increased AD. Per quartile increase of PBDE mixture was associated with a higher risk of depression (OR = 2.93, 95%CI: 1.18, 7.82).
Vuong [64]	Cohort	2003–2006, 8 years follow up	Greater Cincinnati, USA	AD: 83/373, 8-year PPD: 41/223	GC-MS/MS for PBDE (BDE-28, − 47, −99, − 100, −153, and ∑PBDEs), LC-MS/MS for PFAS (PFOA, PFOS, PFHxS, PFNA) using maternal serum at 16 ± 3 weeks’ gestation	PND (BDI-II score > 13) at ~ 20 weeks’ gestation and up to seven times during postpartum visits (4 weeks, 1, 2, 3, 4, 5, and 8 years); used group-based trajectory modeling to predict trajectories of BDI-II scores (low: 49%, medium: 40%, high: 10%)	+: A 10-fold increase in BDE-28 was associated with increased BDI-II scores (β = 2.5 points, 95%CI: 0.8, 4.2) from pregnancy to 8 years postpartum. Significant positive associations were also observed with BDE-47, −100, −153, and ∑PBDEs. A 10-fold increase in ∑PBDEs was associated with a 4.6-fold increased risk (95%CI: 1.8, 11.8) of a high trajectory for BDI-II compared to a low trajectory. **Null**: No significant associations between PFAS and BDI-II scores.

Abbreviations: *PPD* postpartum depression, *AD* antenatal depression, *PND* perinatal depression, *TRI-1* 1 st trimester, *TRI-2* 2nd trimester, *TRI-3* 3rd trimester, *OR* odds ratio, *RR* risk ratio, *CI* confidence interval,* IQR* interquartile range, *EPDS* Edinburgh Postnatal Depression Scale, *CES-D* Center for Epidemiologic Studies Depression Scale, *EDS* Edinburgh Depression Scale, *BDI-II* Beck Depression Inventory-II, *PROMIS* Patient-Reported Measurement Information System, *SPE-LC-MS/MS* solid phase extraction system coupled to liquid chromatography and tandem mass spectrometry, *LC-MS/MS* liquid chromatography and tandem mass spectrometry, *HPLC-QTRAP 5500 + MS/MS* HPLC and coupled with an AB SCIEX QTRAP 5500 + triple quadrupole mass spectrometer, *HPLC-MS/MS* high-performance liquid chromatography-tandem mass spectrometry, *SPE-GC-ECD* solid-phase extraction and gas chromatography with electron capture detector, *LLE-ID-HPLC-MS/MS* liquid-liquid extraction coupled with isotope dilution high-performance liquid chromatography-tandem mass spectrometry, *MS* mass spectrometry, *GC-MS/MS* gas chromatography mass-spectroscopy, *UPLC* system coupled to a TSQ Quantis MS, ultra performance liquid chromatography system coupled to a triple stage quadrupole quantis mass spectrometer, *OPEs* organophosphate ester flame retardants and plasticizers, *OPEs* organophosphate esters, *BBOEP* bis(butoxyethyl) phosphate, *BCEP* bis(2-chloroethyl) phosphate, *BCIPP* bis(1-chloro-2-propyl) phosphate, *BDCIPP* bis(1,3,-dichloro-2-propyl) phosphate, *BEHP* bis(2-ethylhexyl) phosphate, *BMPP* bis(2-methylphenyl) phosphate, *DNBP* di-n-butyl phosphate, *DIBP* di-isobutyl phosphate, *DPHP* diphenyl phosphate, *DPRP* dipropyl phosphate, *OCPs* organochlorine pesticides, *DDT* dichlorodiphenyltrichloroethane, *HCH* hexachlorocyclohexane, *PFAS* per- and polyfluoroalkyl substances, *PFOA* perfluorooctanoate acid, *PFOS* perfluorooctanesulfonate acid, *PFNA* perfluorononanoic acid, *PFDA* perfluorodecanoic acid, *PFUdA* perfluoroundecanoic acid, *PFHxS* perfluorohexanesulfonic acid, *PFHpS* perfluoroheptane sulfonate, *6:2 Cl-PFESA* 6:2 chlorinated perfluoroalkyl ether sulfonate, *PFBS* perfluorobutane sulfonate, *PFHpA* perfluoroheptanoic acid, *PFDeA* perfluorodecanoic acid, *PFDoA* perfluorododecanoic acid, *PFOSA* perfluorooctane sulfonamide, *Me-PFOSA-AcOH* methyl-perfluorooxtane sulfonamide acetic acid, *Et-PFOSA-AcOH* ethyl-perfluorooctane sulfonamide acetic acid, *PFUA* perfluoroundecanoic acid, *LMW* low-molecular-weight, *HMW* high-molecular-weight, *DEHP* di (2-ethylhexyl) phthalate, *BPA* bisphenol A, *BPF* bisphenol F, *BPS* bisphenol S, *BPAF* bisphenol AF, *BPAP* bisphenol AP, *BPB* bisphenol B, *BPP* bisphenol P, *BPPZ* bisphenol PZ, *MeP* methyl paraben, *EtP* ethyl paraben, *PrP* propyl paraben, *MEP* monoethyl phthalate, *MiBP* monoisobutyl phthalate, *MBP* mono-n-butyl phthalate, *MBzP* monobenzyl phthalate, *MEHP* mono-2-ethylhexyl phthalate, *MEHHP* mono (2-ethyl-5-hydroxyhexyl) phthalate, *MEOHP* mono-2-ethyl-5-oxohexyl phthalate, *MECPP* mono-2-ethyl-5-carboxypentyl phthalate, *DnOP* di-n-octyl phthalate, *MEHHP* mono-(2-ethyl-5-hydroxyhexyl) phthalate, *MnBP* mono-N-butyl phthalate, *MCOP* mono-(carboxyoctyl) phthalate, *MiNP* mono-isononyl phthalate, *MP* methylparaben, *EP* ethylparaben, *PP* propylparaben, *TCS* triclosan, *PBDE* polybrominated diphenyl ether.

We noted that two studies were conducted in the CANDLE cohort in Tennessee (Barrett et al., 2022) and the CHES cohort in New York City (Jacobson et al. [60]), respectively, which were two of the five cohorts in the study using U.S. Environmental Influences on Child Health Outcomes (ECHO) consortium (Jacobson et al. [58]).

#### Nonpersistent Organic Pollutants: Phthalates, Phenols, and Parabens

In the six cohort studies evaluated, the nonpersistent chemicals were measured in pregnancy urine samples (*n* = 5) or breast milk (*n* = 1). Each study on phthalates measured ten or more phthalate metabolites. Overall, phthalates have been linked to maternal postpartum depression across the studies included. For example, higher prenatal levels of di‑n‑octyl phthalate (DnOP) and diisononyl phthalate (DiNP) were associated with decreased progesterone and increased odds of postpartum depression [60], and elevated mono‑(2‑ethylhexyl) phthalate (MEHP) in breast milk was also correlated with depressive symptom severity [61]. Phthalate mixtures likewise raised the likelihood of postpartum depression [59]. In contrast, a pooled analysis of the ECHO program reported null associations between pregnancy urinary phthalate markers and maternal postpartum depression overall [58]; however, the study reported that some associations emerged after excluding its largest sub-cohort in the analysis.

No consistent associations were found for phenols or parabens. Higher urinary bisphenol A (BPA) concentrations were linked to increased EPDS depression scores assessed at one month postpartum in a Chinese cohort, particularly, second-trimester exposure [59]. However, associations were inconsistent in other studies for bisphenols [58, 60, 61]. Exposure to triclosan, another type of commonly used phenols, was not associated with postpartum depression in the ECHO program [58] and a Korean cohort study [61]. The studies of parabens reviewed included methyl paraben (MeP), ethyl-paraben (EtP), and propylparaben (PrP). Urinary parabens, modeled individually or as a mixture, were not associated with depressive symptoms in the Chinese study [59] and the ECHO program [58], but a tentative association was reported for EtP in the Korean cohort (*p = 0.091*) [61].

In summary, phthalates emerged as the nonpersistent EDC that were most consistently associated with maternal postpartum depression. Results for phenols and parabens were largely null, with isolated associations reported for bisphenol A in specific antenatal exposure windows and a tentative association for ethyl-paraben in one study. The null findings for some nonpersistent pollutants may not yet exclude the biological plausibility of an effect, as other methodological challenges in measuring nonpersistent pollutants with short biological half-lives also need to be considered.

#### Persistent Organic Pollutants: PFAS, PBDE, OPE, and OCP

Six studies examined blood concentrations of PFAS during pregnancy (*n* = 5) or postpartum (*n* = 1), and their associations with postpartum depression (*n* = 3), antenatal depression (*n* = 2), and antenatal stress (*n* = 1). The five cohort or cross-sectional studies focusing on maternal depression have reported mixed findings: PFAS mixtures increased odds of postpartum depression by 54–75% in a Chinese cohort [55], perfluorooctane sulfonic acid (PFOS) and methyl-perfluorooctane sulfonamide acetic acid (Me-PFOSA-AcOH) were associated with higher depression scores among immigrant mothers in California [56], but no associations were found in a smaller cohort in South Korea [61], a larger cohort in Shanghai [57], and the HOME study in United States that followed maternal depression up to eight years postpartum [64]. These inconsistencies could be due to differences in the characteristics of specific PFAS compounds and their exposure range evaluated [71, 72], and the timing of outcome measures.

Three studies across the United States assessed PBDE exposures from blood during pregnancy, and their links to maternal depression [62–64]. These studies of cohort, case-control, and cross-sectional designs consistently reported increased odds of depression linked to multiple PBDE: BDE-47 and BDE-99 were associated with depressive symptoms at 8–14 weeks gestation (1.58–4.43) [63], each two-fold BDE-47 increase was associated with up to 22% higher odds of ICD-10 depression diagnosis up to one year postpartum [62], and a 10-fold increase in BDE-28 and total PBDEs was linked to elevated BDI-II scores (up to 2.5 points) and high depression trajectory risk (4.6-fold) through eight years postpartum [64]. The findings in the pregnant women overall corroborate with recent findings in a meta-analysis of adult depression that showed exposure to total PBDE, BDE-47, and BDE-99 were linked to increased risk of depression, but not for other PBDE congeners, such as BDE-28, BDE-100, and BDE-153 [73].

Two studies in the USA and Canada investigated OPE flame retardants and their associations with CES-D depression scores [52, 53]. Of the 10 OPE metabolites assessed in the third-trimester single spot urine in the USA cohort, the highest tertiles of diphenyl phosphate (DPHP) and bis(1,3-dichloro-2-propyl) phosphate (BDCIPP) levels were associated with increased odds of depressive symptoms by 67% and 47%, respectively, with the OPE mixtures showing a linear effect [53]. However, the total OPE Z-score of 29 OPE measured in the house dust during the 3–4-month postpartum period was not associated with depressive symptoms in the Canadian cohort [52].

While pesticides are known to affect multiple neurological disorders [70], we found only one study examining 12 OCP in breast milk and their relationships with maternal psychopathologies from delivery to eight months postpartum in a cohort of 75 lactating women from suburban Ankara, Turkey [54]. The Spearman’s correlation analyses did not find associations between the OCP markers and EPDS scores.

Overall, evidence for persistent organic pollutants varied by chemical class. While the number of studies remains limited, PBDE exposures were associated with maternal depression from early pregnancy through long-term postpartum follow-up. Findings for PFAS were inconclusive, and the evidence for OPE flame retardants and organochlorine pesticides was sparse.

### Metals

Studies of metals used diverse types of biological samples for exposure assessments, including blood, urine, and toenails collected at different trimesters of pregnancy or at delivery, as well as Tibia (cortical bone) and patella (trabecular bone) lead measurements with an X-ray fluorescence around one month postpartum.

Lead was the most investigated metal in this research area, with three focusing on depression assessed by EPDS (Table 3). The Project Viva [76] of 1226 women evaluated joint exposure to 11 metals (arsenic, barium, cadmium, cesium, copper, mercury, magnesium, manganese, lead, selenium, and zinc) in first-trimester erythrocytes using Bayesian kernel machine regression (BKMR) [77]. The study reported no associations between the metal mixture and EPDS scores, while imprecise positive associations were noted between three metals (arsenic, lead, and selenium) and higher odds of depressive symptoms at mid-pregnancy or postpartum (EPDS score > = 13). The PROGRESS cohort [74] of 561 women in Mexico City investigated the relationship between repeated blood manganese measurements and EPDS scores at 12 months postpartum, and reported a positive association for blood manganese measured at the third-trimester only, but not at the second trimester or delivery. In a cohort study concerning arsenic exposure conducted in 2013 of 223 women from Arica, Chile, urinary inorganic arsenic was inversely associated with EPDS scores in women older than 25 years without a history of depression [75].

**Table 3:** Summary of articles examining the associations between metals and maternal depression

Citation	Study design	Study year	Location	Sample size	Exposure measurement	Outcome measurement	Key findings
Rokoff [76]	Cohort	1999–2002	Eastern Massachusetts, USA	During pregnancy: 96/1226, 6 months postpartum: 75/1226, 12 months postpartum: 53/1226	ICP-MS for 11 metals (As, Ba, Cd, Cs, Cu, Hg, Mg, Mn, Pb, Se, Zn) and mixture using blood samples collected in early pregnancy (median 9.6 weeks ± 2.2)	PND (EPDS score > = 13) at mid-pregnancy and at 6 and 12 months postpartum; used latent class mixed modeling to model trajectories of depressive symptoms (continuous EPDS scores)	**Null**: The early pregnancy erythrocyte metal mixture was not associated with maternal depressive symptoms. For individual metals, most CI included null. +: There was weak evidence that As, Pb, and Se were moderately associated with elevated odds of depressive symptoms and/or trajectories. **However**, the ORs (95%CI) per doubling of these three metals were imprecise [e.g., As: OR = 1.13, 95%CI: 0.94, 1.40 for 6 months PPD; Pb: OR = 1.19, 95%CI: 0.80, 1.77 for AD at mid-pregnancy; Se: OR = 2.35, 95%CI: 0.84, 6.57 for elevated mid-pregnancy, then decreasing versus stable low trajectory].
McRae [74]	Cohort	Jul 2007–Feb 2011	Mexico City, Mexico	NA/561	ICP-DRC-MS for Mn using blood samples measured at TRI-2, TRI-3, and at delivery	PPD (continuous EPDS scores) at 12 months postpartum	+: TRI-3 BMn (β = 0.13, 95% CI: 0.04, 0.21) and BMn levels averaged at the 2nd and 3rd trimester (β = 0.14, 95% CI: 0.02, 0.26) had a positive association with 12 months PPD; **Null**: TRI-2 BMn (β = 0.07, 95% CI: −0.09, 0.22) and delivery (β = 0.03, 95% CI: −0.04, 0.10) had a non-significant positive association with 12-months PPD.
Valdés [75]	Cohort	Jun–Oct 2013	Arica, Chile	46/223	HPLC-ICPMS for arsenic using urine samples collected during the morning after recruitment	PPD (continuous EPDS scores) at 3–12 months postpartum	**Null**: In women aged 25 years or younger, with and without depression history, there was no significant association between Ln iAs and PPD. -: For women older than 25 years old without depression history, the Ln iAs was protective (β=−2.51, 95%CI: −4.54, − 0.48). +: For women older than 25 years old with a depression history, Ln iAs and PPD were not statistically associated (β = 2.09, 95%CI: −0.90, 5.08).

Abbreviations: *PPD* postpartum depression, *AD* antenatal depression, *TRI-2* 2nd trimester, *TRI-3* 3rd trimester, *OR* odds ratio, *CI* confidence interval, *IQR* interquartile range, *ICP-MS* inductively-coupled plasma mass spectrometry, *ICP-DRC-MS* inductively coupled plasma mass spectrometer, *HPLC-ICPMS* high-performance liquid chromatography inductively coupled plasma mass spectrometry, *As* arsenic, *Ba* barium, *Cd* cadmium, *Cs* cesium, *Cu* copper, *Hg* mercury, *Mg* magnesium, *Mn* manganese, *Pb* lead, *Se* selenium, *Zn* zinc, *iAs* inorganic arsenic, *BMn* blood manganese, *EPDS* Edinburgh Postnatal Depression Scale, *Ln* natural logarithm

Overall, the small number of studies and heterogeneous exposure assessment and timing preclude firm conclusions regarding the relationship between metals and maternal depression. Nonetheless, some imprecise or exposure-window-specific signals reported for select metals (e.g., arsenic, lead, manganese, selenium) warrant further investigation.

## Future Directions

### Broadening the Scope of Chemicals

Through our scoping review, we have identified research gaps and priorities for future investigations. First, roughly one-third of the existing studies focused on ambient air pollution, and studies on other environmental contaminants, including different types of EDC and metals, remained sparse. While exposures to traffic- and industry-related NO_2_ and PM_10_ in outdoor air have been linked to maternal depression, research that explores personal exposures, pollution sources and chemical species, the biologic effects, and the interplay with individual susceptibility and climate factors are needed. Findings for other types of chemicals, including various EDC and metals, are not yet conclusive and need further evidence, preferably using larger study sample sizes with valid instruments that capture pregnancy and postpartum depression. Other widespread or emerging contaminants from water, food, or personal products that have neurologic effects or neuroinflammatory potentials, such as pesticides and microplastics, may also need to be scrutinized.

### Improving Assessment of Maternal Depression

Second, assessment of maternal depression in the environmental studies needs refinement. Perinatal depressive symptoms can begin antenatally and persist for 3 years postpartum [78], while most studies have relied on a single assessment between 4 weeks and 12 months after delivery. Administering repeated validated screening instruments prospectively from early gestation through years after birth should be considered, but balancing participation burden is needed [79]. Large-scale electronic health records or registry data, indexed by diagnostic or prescription codes, offer a cost-effective alternative that captures the onset and persistence of maternal depression [80], but studying diagnostic codes may lack details on symptoms, severity, and pre-clinical outcomes. Without a gold standard method to measure maternal depression, multiple assessment tools may need to be used to triangulate the research findings [81]. In addition to screening tools and medical records, biomarker approaches indicative of mental health [13], such as HPA-axis and dopaminergic signatures [13], oxytocin-pathway genotype–epigenetic interactions [82], and neuroimaging markers [83], can also be explored. Improvements in screening and detection of perinatal depression would help advance etiology research, including the search for environmental risk factors.

### Addressing Mixtures and Multifactorial Etiology

Third, depressive disorders are known to have multifactorial origins, which prompt the consideration of studies that examine the joint effects across various chemical exposures (e.g., the mixture), as well as the potential interaction of the environmental chemicals with other non-chemical stressors such as nutritional, lifestyle, social, and genetic risk factors [47, 84–86]. Future studies should pursue advanced statistical modeling to estimate the joint effects of chemical mixtures. Study has reported that polymorphisms in the oxytocin pathway can worsen depressive symptoms under financial strain or poor family relationships [11], yet analogous gene-environment interactions for chemical exposures remain largely untested. Some nutritional or lifestyle factors can either accelerate or counterbalance the effects of environmental chemicals on pregnancy or birth outcomes [87, 88], and particularly the modifiable effect modifiers should be considered when studying perinatal depression. For example, breastfeeding can facilitate the excretion of some environmental chemicals from the mother, while maternal exposures may also contribute to breastfeeding difficulties that are interrelated with postpartum depression or emotional stress [89]. Maternal PFAS burdens are suspected to interfere with mammary gland development and reduce breastfeeding duration, plausibly diminishing prolactin/oxytocin mood-buffering and amplifying the risk of maternal depression [90]. Ultimately, an exposome approach with the inclusion of techniques that map the totality of exposures from diverse aspects and across time, including diet, behaviors, toxicology, social stress, working environment, and neighborhood characteristics [91, 92], would be helpful to reveal the complexity of the effects of cumulative and multiple exposures.

### Addressing Selection Bias

Fourth, potential selection biases in the analyses of exposure effects and maternal mental health need attention. Maternal preconception of mental illness may reduce fertility and increase the risk of severe maternal morbidity, thus the likelihood of being enrolled in a pregnancy cohort study [93, 94]. Similarly, maternal mental disorder is also a strong risk factor for pregnancy loss, stillbirth, neonatal and infant mortality, which leads to censoring in the birth cohort [95]. Thus, even record-based studies without any contact with the study participants could end up with a selective subpopulation for statistical analyses [96]. Further, individuals who develop mental health conditions and/or have a disadvantaged background are less likely to join or be retained in studies that require in-person participation or self-reports [97, 98], inducing self-selection or loss-to-follow up bias. Epidemiologic and statistical methods that can be used to evaluate and adjust for these potential selection biases need to be considered [99–102].

### Addressing Confounding and Exposure Measurement Error

Further, studies that address potential unmeasured or residual confounding bias and exposure measurement error are also needed. Socioeconomic, psychosocial, and clinical factors are strong determinants of both environmental exposures and maternal depression, and the confounding roles for maternal and family history of mental disorder, stressful life events, occupation, and chronic health conditions in the current literature have not been resolved [103]. In addition, exposure misclassification remains a concern. Many studies in the current literature did not adequately capture the entire etiologically relevant exposure windows for depression during pregnancy and postpartum, for example, by relying on spot biospecimen measurements to assess nonpersistent chemicals. Depending on the structure of the uncontrolled confounding and measurement error, these issues may bias the associations toward or away from the null. Improved study designs, including novel approaches [104, 105], along with advanced analytical methods to better address confounding and exposure assessment are essential to strengthen causal inference.

## Limitations

Our review has several limitations. First, studies included in this review were heterogeneous in terms of exposure and outcome assessment instruments, which may explain inconsistencies in findings and hinder a formal systematic review at this stage. Given the range of studies captured, our manuscript focused on summarizing studies that detailed the environmental chemicals examined and on depression, while information for the non-discussed studies is provided in supplementary files. Second, we did not evaluate the strength of evidence or study quality in this scoping review. Although written language was not an exclusion criterion, articles written in a non-English language may be less likely to be captured in our English search terms. Third, publication bias cannot be ruled out if positive findings are more likely to be published in this field.

## Conclusions

Environmental exposures, including certain airborne pollutants and chemicals from contaminated household products and food sources, may contribute to the development of maternal depression during and after pregnancy. Continued investigation of environmental influences on maternal depression is warranted to determine whether environmental risk reduction is an effective strategy to inform primary prevention of maternal depression.

##  Key References

- Slomian J, Honvo G, Emonts P, Reginster J-Y, Bruyère O. Consequences of maternal postpartum depression: A systematic review of maternal and infant outcomes. Womens Health. 2019;15:1745506519844044.
  - ◌ A systematic review summarizing the wide-ranging consequences of maternal postpartum depression for both mothers and infants.
- Payne JL, Maguire J. Pathophysiological mechanisms implicated in postpartum depression. Front Neuroendocrinol. 2019;52:165–180.
  - ◌ This review paper discusses neuroendocrine, inflammatory, and genetic mechanisms contributing to the pathophysiology of postpartum depression.
- Yu Y, Liang HF, Chen J, Li ZB, Han YS, Chen JX, Li JC. Postpartum Depression: Current Status and Possible Identification Using Biomarkers. Front Psychiatry. 2021;12:620371.
  - ◌ An overview of the epidemiology and clinical presentation of postpartum depression, emphasizing the potential role of biomarkers in early detection.
- Jacobson MH, Ghassabian A, Gore AC, Trasande L. Exposure to environmental chemicals and perinatal psychopathology. Biochem Pharmacol. 2022;195:114835.
  - ◌ A review linking exposure to environmental chemicals with maternal perinatal psychopathology, highlighting evidence among animal models and humans.

## Supplementary Information

Below is the link to the electronic supplementary material.

Supplementary Material 1 (XLSX 104 KB)

Supplementary Material 2 (DOCX 99.5 KB)

## Data Availability

No datasets were generated or analysed during the current study.
