# Supplementary material for: Environmental Chemicals and Maternal Depression During and After Pregnancy: a Scoping Review
Source: Curr Environ Health Rep. 2026 Feb 19;13(1):7. doi: 10.1007/s40572-026-00529-7 (PMC12920714; doi:10.1007/s40572-026-00529-7)
Supplement: Supplementary file 2 — Supplementary Material 2 (DOCX 99.5 KB) [file 40572_2026_529_MOESM2_ESM.docx]

**Appendix A**

#### Pubmed:

(prenatal[tw] OR gestational[tw] OR antenatal[tw] OR perinatal[tw] OR postpartum[tw] OR peripartum[tw] OR pregnan*[tw] OR maternal[tw] OR "Mothers/psychology"[mh] OR "Peripartum Period"[mh] OR "Postpartum Period"[mh] OR "Maternal Health"[mh]) AND (stress*[tw] OR anxiet*[tw] OR anxious[tw] OR distress[tw] OR depressi*[tw] OR mental*[tw] OR suicid*[tw] OR “Depressive Disorder”[mh] OR "Emotions"[mh] OR "Self-Injurious Behavior"[mh] OR “Stress, Psychological”[mh]) AND ("chemical exposure"[tiab:~5] OR "chemical exposures"[tiab:~5] OR "chemicals exposure"[tiab:~5] OR "chemicals exposures"[tiab:~5] OR "compound exposure"[tiab:~5] OR "compound exposures"[tiab:~5] OR "compounds exposure"[tiab:~5] OR "compounds exposures"[tiab:~5] OR “environmental exposures”[tiab:~5] OR “environmental exposure”[tiab:~5] OR “environmental chemicals”[tiab:~5] OR “environmental chemical”[tiab:~5] OR “environmental toxicants”[tiab:~5] OR “environmental toxicant”[tiab:~5] OR “environmental pollutants”[tiab:~5] OR “environmental pollutant”[tiab:~5] OR “environmental pollution”[tiab:~5] OR “environmental pollutions”[tiab:~5] OR “toxicant exposure”[tiab:~5] OR “toxicant exposures”[tiab:~5] OR “toxicants exposure”[tiab:~5] OR “toxicants exposures”[tiab:~5] OR “pollutant exposure”[tiab:~5] OR “pollutant exposures”[tiab:~5] OR “pollutants exposure”[tiab:~5] OR “pollutants exposures”[tiab:~5] OR xenobiotic*[tw] OR “industrial waste*”[tw] OR “metal exposure”[tiab:~5] OR “metal exposures”[tiab:~5] OR “metals exposure”[tiab:~5] OR “metals exposures”[tiab:~5] OR “endocrine disrupt*”[tw] OR pesticide*[tw] OR bisphenol[tw] OR BPA[tw] OR PCB[tw] OR PCBs[tw] OR “polychlorinated biphenyl*”[tw] OR PFAS*[tw] OR perfluoro*[tw] OR polyfluoro*[tw] OR PFOS[tw] OR PFOA[tw] OR PFNA[tw] OR PFHxS[tw] OR “phthalate*”[tw] OR plasticizer*[tw] OR “phthalic acid*”[tw] OR PBDE*[tw] OR “polybrominated diphenyl*”[tw] OR “polycyclic aromatic hydrocarbons”[tw] OR “polyaromatic hydrocarbons”[tw] OR PAHs[tw] OR “tobacco smok*”[tw] OR “secondhand smok*”[tw] OR “passive smok*”[tw] OR organophosphate*[tw] OR mixture*[tw] OR “Environmental Pollution”[mh] OR "Environmental Pollutants"[mh] OR “Endocrine Disruptors”[mh] OR "Xenobiotics"[mh] OR "Hazardous Substances"[mh] OR "Pesticides"[mh] OR "Organic Chemicals"[mh] OR "Inorganic Chemicals"[mh] OR "Complex Mixtures"[mh] OR "Specialty Uses of Chemicals"[mh]) NOT (animal[mh] NOT humans[mh]) NOT (review[ti] OR overview[ti] OR review[pt])

#### Dimensions:

search in full data, restrict document type to research articles

(title:prenatal OR title:gestational OR title:antenatal OR title:perinatal OR title:postpartum OR title:peripartum OR title:pregnan* OR title:maternal OR abstract:prenatal OR abstract:gestational OR abstract:antenatal OR abstract:perinatal OR abstract:postpartum OR abstract:peripartum OR abstract:pregnan* OR abstract:maternal OR mesh_terms:(Mothers/psychology) OR mesh_terms:(Peripartum Period) OR mesh_terms:(Postpartum Period) OR mesh_terms:(Maternal Health)) AND (title:stress* OR title:anxiet* OR title:anxious OR title:distress OR title:depressi* OR title:mental* OR title:suicid* OR abstract:stress* OR abstract:anxiet* OR abstract:anxious OR abstract:distress OR abstract:depressi* OR abstract:mental* OR abstract:suicid* OR mesh_terms:(Depressive Disorder) OR mesh_terms:(Emotions) OR mesh_terms:(Self-Injurious Behavior) OR mesh_terms:(Stress, Psychological)) AND (“chemical exposure"~5 OR "chemical exposures"~5 OR "chemicals exposure"~5 OR "chemicals exposures"~5 OR "compound exposure"~5 OR "compound exposures"~5 OR "compounds exposure"~5 OR "compounds exposures"~5 OR “environmental exposures”~5 OR “environmental exposure”~5 OR “environmental chemicals”~5 OR “environmental chemical”~5 OR “environmental toxicants”~5 OR “environmental toxicant”~5 OR “environmental pollutants”~5 OR “environmental pollutant”~5 OR “environmental pollution”~5 OR “environmental pollutions”~5 OR “toxicant exposure”~5 OR “toxicant exposures”~5 OR “toxicants exposure”~5 OR “toxicants exposures”~5 OR “pollutant exposure”~5 OR “pollutant exposures”~5 OR “pollutants exposure”~5 OR “pollutants exposures”~5 OR xenobiotic* OR “industrial waste*” OR “metal exposure”~5 OR “metal exposures”~5 OR “metals exposure”~5 OR “metals exposures”~5 OR title:(endocrine disrupt*) OR title:pesticide* OR title:bisphenol OR title:BPA OR title:PCB OR title:PCBs OR title:(polychlorinated biphenyl*) OR title:PFAS* OR title:perfluoro* OR title:polyfluoro* OR title:PFOS OR title:PFOA OR title:PFNA OR title:PFHxS OR title:phthalate* OR title:plasticizer* OR title:(phthalic acid*) OR title:PBDE* OR title:(polybrominated diphenyl*) OR title:(polycyclic aromatic hydrocarbons) OR title:(polyaromatic hydrocarbons) OR title:PAHs OR title:(tobacco smok*) OR title:(secondhand smok*) OR title:(passive smok*) OR title:organophosphate* OR title:mixture* OR abstract:(endocrine disrupt*) OR abstract:pesticide* OR abstract:bisphenol OR abstract:BPA OR abstract:PCB OR abstract:PCBs OR abstract:(polychlorinated biphenyl*) OR abstract:PFAS* OR abstract:perfluoro* OR abstract:polyfluoro* OR abstract:PFOS OR abstract:PFOA OR abstract:PFNA OR abstract:PFHxS OR abstract:phthalate* OR abstract:plasticizer* OR abstract:(phthalic acid*) OR abstract:PBDE* OR abstract:(polybrominated diphenyl*) OR abstract:(polycyclic aromatic hydrocarbons) OR abstract:(polyaromatic hydrocarbons) OR abstract:PAHs OR abstract:(tobacco smok*) OR abstract:(secondhand smok*) OR abstract:(passive smok*) OR abstract:organophosphate* OR abstract:mixture* OR mesh_terms:(Environmental Pollution) OR mesh_terms:(Environmental Pollutants) OR mesh_terms:(Endocrine Disruptors) OR mesh_terms:(Xenobiotics) OR mesh_terms:(Hazardous Substances) OR mesh_terms:(Pesticides) OR mesh_terms:(Organic Chemicals) OR mesh_terms:(Inorganic Chemicals) OR mesh_terms:(Complex Mixtures) OR mesh_terms:(Specialty Uses of Chemicals)) NOT (mesh_terms:(animals) NOT mesh_terms:(humans))

#### Web of Science Core Collection as licensed at Yale:

TS = ((prenatal OR gestational OR antenatal OR perinatal OR postpartum OR peripartum OR pregnan* OR maternal OR "Mothers/psychology" OR "Peripartum Period" OR "Postpartum Period" OR "Maternal Health") AND (stress* OR anxiet* OR anxious OR distress OR depressi* OR mental* OR suicid* OR “Depressive Disorder” OR "Emotions" OR "Self-Injurious Behavior" OR “Stress, Psychological”) AND ((chemical* NEAR/5 exposure*) OR (compound* NEAR/5 exposure*) OR (environmental NEAR/5 exposure*) OR (environmental NEAR/5 chemical*) OR (environmental NEAR/5 toxicant*) OR (environmental NEAR/5 pollut*) OR (toxicant* NEAR/5 exposure*) OR  (pollut* NEAR/5 exposure*) OR xenobiotic* OR “industrial waste*” OR (metal* NEAR/5 exposure*) OR “endocrine disrupt*” OR pesticide* OR bisphenol OR BPA OR PCB OR PCBs OR “polychlorinated biphenyl*” OR PFAS* OR perfluoro* OR polyfluoro* OR PFOS OR PFOA OR PFNA OR PFHxS OR “phthalate*” OR plasticizer* OR “phthalic acid*” OR PBDE* OR “polybrominated diphenyl*” OR “polycyclic aromatic hydrocarbons” OR “polyaromatic hydrocarbons” OR PAHs OR “tobacco smok*” OR “secondhand smok*” OR “passive smok*” OR organophosphate* OR mixture* OR “Environmental Pollution” OR "Hazardous Substances" OR "Organic Chemicals" OR "Inorganic Chemicals" OR "Specialty Uses of Chemicals"))

The databases linked through the Web of Science (WOS) as licensed at Yale were:

#### Web of Science Core Collection: Citation Indexes

- Science Citation Index Expanded (SCI-EXPANDED) --1900-present
- Social Sciences Citation Index (SSCI) --1900-present
- Arts & Humanities Citation Index (A&HCI) --1975-present
- Conference Proceedings Citation Index- Science (CPCI-S) --1991-present
- Conference Proceedings Citation Index- Social Science & Humanities (CPCI-SSH) --1991-present
- Book Citation Index– Science (BKCI-S) --2005-present
- Book Citation Index– Social Sciences & Humanities (BKCI-SSH) --2005-present
- Emerging Sources Citation Index (ESCI) --2015-present

#### Web of Science Core Collection: Chemical Indexes

- Current Chemical Reactions (CCR-EXPANDED) --1985-present
  *(Includes Institut National de la Propriete Industrielle structure data back to 1840)*
- Index Chemicus (IC) --1993-present

***Embase via Ovid***

| **#** | **Query** |
| --- | --- |
| **1** | [Pregnancy-related final] |
| **2** | exp prenatal exposure/ or exp prenatal period/ or exp prenatal stress/ or exp pregnancy/ or exp antenatal depression/ or exp perinatal depression/ or exp perinatal period/ or exp perinatal exposure/ or exp maternal exposure/ |
| **3** | (prenatal or gestational or antenatal or perinatal or postpartum or peripartum or pregnan* or maternal).mp. |
| **4** | 2 or 3 |
| **5** | [Mental-related final] |
| **6** | exp physiological stress/ or exp anxiety/ or exp anxiety disorder/ or exp "Depression, Anxiety and Stress Scale"/ or exp distress syndrome/ or exp major depression/ or exp depression/ or exp postnatal depression/ or exp Edinburgh Postnatal Depression Scale/ or exp mental stress/ or exp mental health/ or exp mental disease/ |
| **7** | (stress or anxiet* or anxious or distress or depressi* or mental* or suicid*).mp. |
| **8** | 6 or 7 |
| **9** | [Chemical exposure-related final] |
| **10** | ((chemical* or compound* or environmental* or toxicant* or pollut* or metal*) adj5 exposure*).mp. |
| **11** | (environmental adj5 (chemical* or toxicant* or pollut*)).mp. |
| **12** | (xenobiotic or "industrial waste" or "endocrine disrupt*" or pesticide or bisphenol or bpa or pcb or "polychlorinated biphenyl*" or pfas or perfluoro* or polyfluoro* or pfos or pfoa or pfna or pfhxs or "phthalate" or plasticizer or "phthalic acid" or pbde or "polybrominated diphenyl*" or "polycyclic aromatic hydrocarbon" or "polyaromatic hydrocarbon" or pah or "tobacco smok*" or "secondhand smok*" or "passive smok*" or organophosphate or mixture or "Environmental Pollution" or "Hazardous Substances" or "Organic Chemicals" or "Inorganic Chemicals" or "Specialty Uses of Chemicals").mp. |
| **13** | 10 or 11 or 12 |
| **14** | 4 and 8 and 13 |
| **15** | limit 14 to (human and "remove preprint records" and article and journal) |

***Scopus***

TITLE-ABS-KEY ( prenatal OR gestational OR antenatal OR perinatal OR postpartum OR peripartum OR pregnan* OR maternal OR "Mothers/psychology" OR "Peripartum Period" OR "Postpartum Period" OR "Maternal Health" ) AND ( stress OR anxiet* OR anxious OR distress OR depressi* OR mental* OR suicid* OR "Depressive Disorder" OR "Emotions" OR "Self-Injurious Behavior" OR "Stress, Psychological" ) AND ( ( ( chemical OR compound OR environmental OR toxicant OR pollut* OR metal ) W/5 exposure ) OR ( environmental W/5 ( chemical OR toxicant OR pollut* ) ) OR xenobiotic OR "industrial waste" OR "endocrine disrupt*" OR pesticide OR bisphenol OR bpa OR pcb OR "polychlorinated biphenyl*" OR pfas OR perfluoro* OR polyfluoro* OR pfos OR pfoa OR pfna OR pfhxs OR "phthalate" OR plasticizer OR "phthalic acid" OR pbde OR "polybrominated diphenyl*" OR "polycyclic aromatic hydrocarbon" OR "polyaromatic hydrocarbon" OR pah OR "tobacco smok*" OR "secondhand smok*" OR "passive smok*" OR organophosphate OR mixture OR "Environmental Pollution" OR "Hazardous Substances" OR "Organic Chemicals" OR "Inorganic Chemicals" OR "Specialty Uses of Chemicals" ) AND ( LIMIT-TO ( SRCTYPE , "j" ) ) AND ( LIMIT-TO ( PUBSTAGE , "final" ) ) AND ( LIMIT-TO ( SUBJAREA , "MEDI" ) OR LIMIT-TO ( SUBJAREA , "ENVI" ) OR LIMIT-TO ( SUBJAREA , "PSYC" ) OR EXCLUDE ( SUBJAREA , "ENGI" ) OR EXCLUDE ( SUBJAREA , "MATH" ) OR EXCLUDE ( SUBJAREA , "DENT" ) OR EXCLUDE ( SUBJAREA , "MATE" ) OR EXCLUDE ( SUBJAREA , "BUSI" ) OR EXCLUDE ( SUBJAREA , "PHYS" ) OR EXCLUDE ( SUBJAREA , "VETE" ) OR EXCLUDE ( SUBJAREA , "ECON" ) OR EXCLUDE ( SUBJAREA , "ENER" ) OR EXCLUDE ( SUBJAREA , "COMP" ) OR EXCLUDE ( SUBJAREA , "ARTS" ) OR EXCLUDE ( SUBJAREA , "CENG" ) ) AND ( EXCLUDE ( EXACTKEYWORD , "Alcohol" ) OR EXCLUDE ( EXACTKEYWORD , "Alcohol Consumption" ) OR EXCLUDE ( EXACTKEYWORD , "Animal" ) OR EXCLUDE ( EXACTKEYWORD , "Animal Cell" ) OR EXCLUDE ( EXACTKEYWORD , "Animal Experiment" ) OR EXCLUDE ( EXACTKEYWORD , "Animal Model" ) OR EXCLUDE ( EXACTKEYWORD , "Animal Tissue" ) OR EXCLUDE ( EXACTKEYWORD , "Animals" ) OR EXCLUDE ( EXACTKEYWORD , "Autism" ) OR EXCLUDE ( EXACTKEYWORD , "Asthma" ) OR EXCLUDE ( EXACTKEYWORD , "Human Cell" ) OR EXCLUDE ( EXACTKEYWORD , "Human Tissue" ) OR EXCLUDE ( EXACTKEYWORD , "Mice" ) OR EXCLUDE ( EXACTKEYWORD , "Nonhuman" ) OR EXCLUDE ( EXACTKEYWORD , "Rat" ) OR EXCLUDE ( EXACTKEYWORD , "Rats" ) OR EXCLUDE ( EXACTKEYWORD , "Signal Transduction" ) OR EXCLUDE ( EXACTKEYWORD , "Unclassified Drug" ) OR EXCLUDE ( EXACTKEYWORD , "Procedures" ) OR EXCLUDE ( EXACTKEYWORD , "Randomized Controlled Trial" ) OR EXCLUDE ( EXACTKEYWORD , "Human Experiment" ) ) AND ( LIMIT-TO ( DOCTYPE , "ar" ) )

*Last access date: Aug 16, 2024*


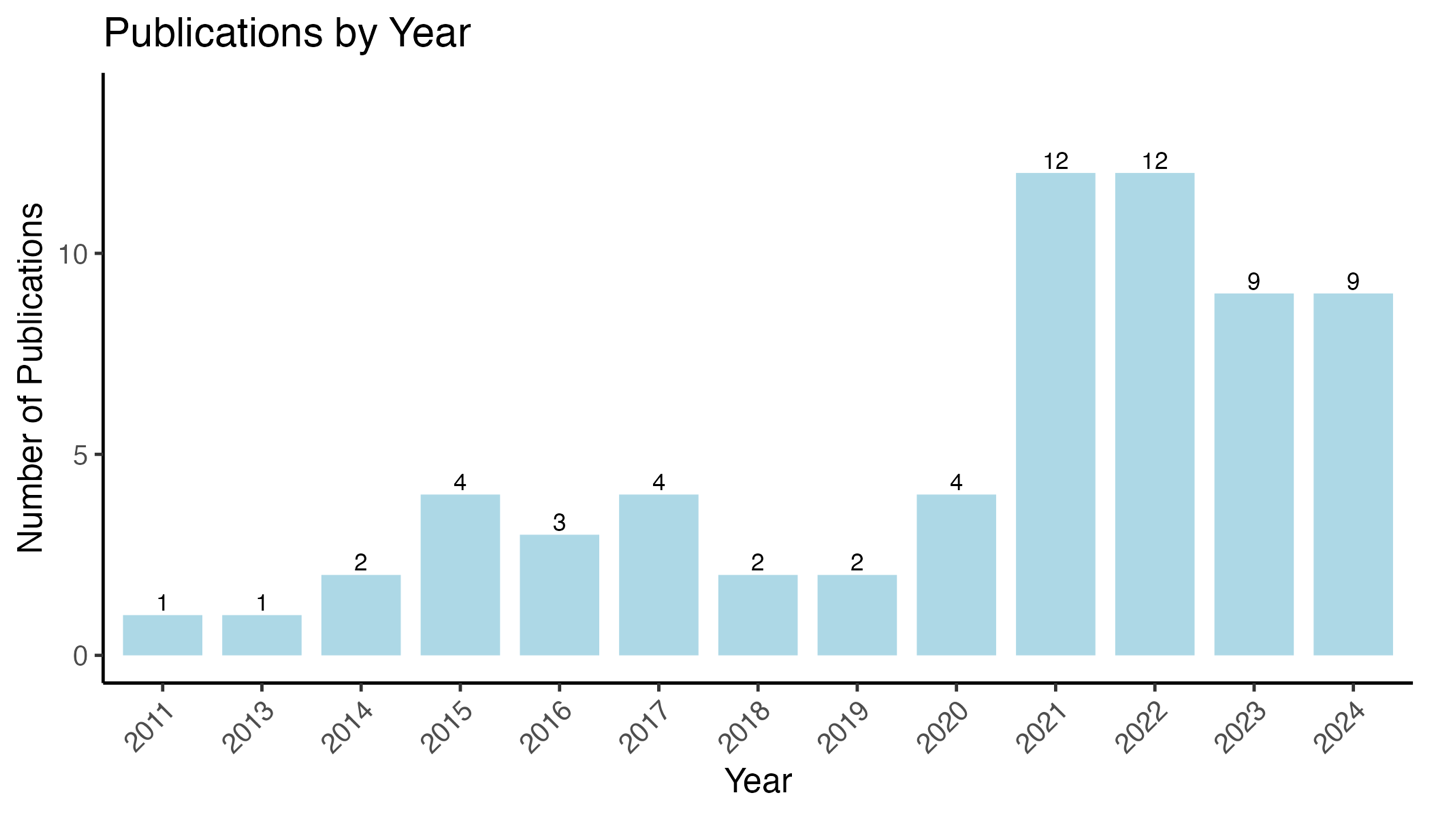


**Figure S1. Distribution of included studies by year of publication (n = 65).**
